# Supplementary material for: GeneSegNet: a deep learning framework for cell segmentation by integrating gene expression and imaging
Source: Genome Biol. 2023 Oct 19;24:235. doi: 10.1186/s13059-023-03054-0 (PMC10585768; doi:10.1186/s13059-023-03054-0)
Supplement: Supplementary file 1 — Additional file 1: Figure S1. Model generalizability comparison of GeneSegNet and Cellpose. Figure S2. Examples of ground truth cell segmentation in the NSCLC dataset. Table S1. Running time and memory usage of GeneSegNet. Table S2. Notation Table. Table S3. The ablation study for the choice of hyper-parameters in the simulation dataset. Table S4. The ablation study for the choice of hyper-parameters in the NSCLC dataset. Table S5. The ablation study for the choice of hyper-parameters in the hippocampus dataset. Figure S3. Overview of the process calculating the reproducibility of gene-gene correlations. [file 13059_2023_3054_MOESM1_ESM.pdf]

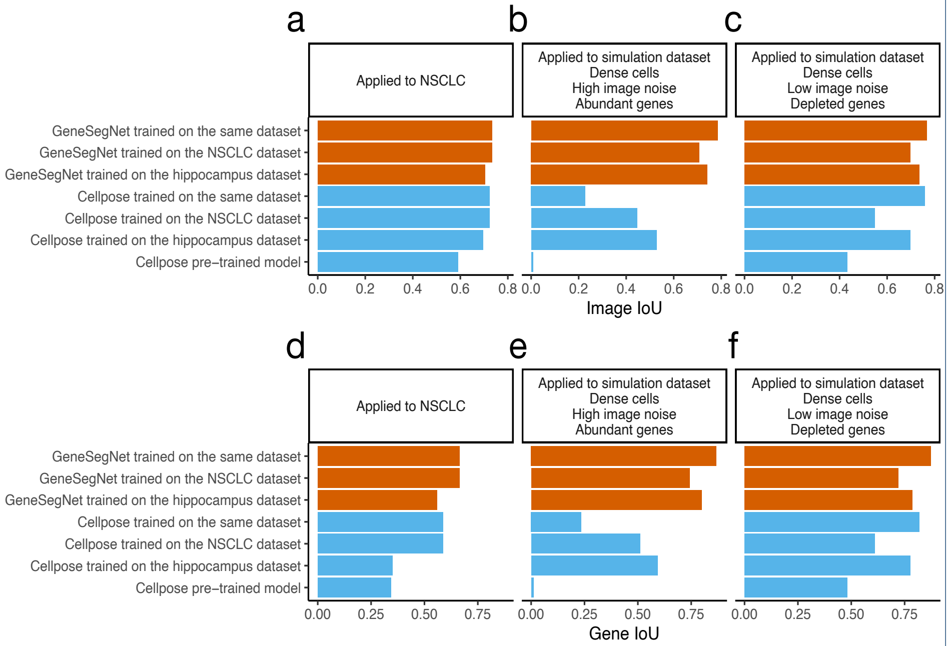

Fig. S1: Image IoU scores (**a-c**) and Gene IoU scores (**d-f**) when GeneSegNet and Cellpose were trained on the same or different datasets as the dataset being applied. **a,d**: Models applied to NSCLC dataset. **b,e**: Models applied to simulation dataset with densely distributed cells, high image noise, and abundant amount of genes. **c,f**: Models applied to simulation dataset with densely distributed cells, low image noise, and depleted amount of genes.

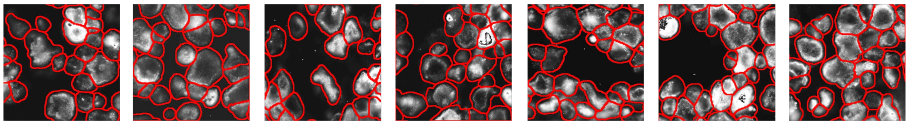

Fig. S2: Examples of ground truth cell segmentation derived by Cellpose using both nuclear and membrane markers in the NSCLC dataset. The detected cell boundaries are marked in red, shown on membrane images as background.

Table S1: Running time and memory usage of GeneSegNet.

Dataset	GeneSegNet Stage	Runtime	Memory Usage	Configuration
Simulation (one scenario)	Training	330 Min	8GB GPU Memory	Image Size = 256 × 256, Epoch = 500, Batch Size = 8, Training and Validation Samples = 344 + 100
NSCLC		720 Min		Image Size = 256 × 256, Epoch = 500, Batch Size = 8, Training and Validation Samples = 961 + 290
Hippocampus		1140 Min		Image Size = 256 × 256, Epoch = 500, Batch Size = 8, Training and Validation Samples = 2098 + 420
Simulation (one scenario)	Inference	20.0 Min	20GB CPU Memory	Image Size = 7248 × 3624
NSCLC		15.0 Min	10GB CPU Memory	Image Size = 5472 × 3648
Hippocampus		20.0 Min	30GB CPU Memory	Image Size = 6130 × 5548

Table S2: Notation Table

Notation	Description
I	input (patch) image of $W \times H$ resolution, $I \in [0,1]^{W \times H}$
g_l	coordinate of the l-th RNA in the lattice of I, $g_l \in \{1, \cdots, W\} \times \{1, \cdots, H\}$
G	input RNA location map, $G \in [0,1]^{W \times H}$
$\boldsymbol{I}$	set of a total of N training (patch) images, $\boldsymbol{I} = \{I_n\}_{n=1}^N$
$\boldsymbol{g}$	set of coordinates of a total of L RNAs in image I, $\boldsymbol{g} = \{g_l\}_{l=1}^L$
$\boldsymbol{G}$	set of a total of N RNA location maps of the training images $\boldsymbol{I}$, $\boldsymbol{G} = \{G_n\}_{n=1}^N$
$f(;\theta)$	neural network of GeneSegNet parameterized by θ
$\hat{M}/M$	network estimation/training label for the confidence map, $\hat{M} \in [0,1]^{W \times H}$, $M \in \{0,1\}^{W \times H}$
$\hat{C}/C$	network estimation/training label for the center map, $\hat{C} \in [0,1]^{W \times H}$, $C \in \{0,1\}^{W \times H}$
$\hat{V}/V$	network estimation/training label for the offset map, $\hat{V}, V \in \mathbb{R}^{2 \times W \times H}$
$\hat{\boldsymbol{Y}}/\boldsymbol{Y}$	set of network outputs/training labels for the training images $\boldsymbol{I}$, $\hat{\boldsymbol{Y}} = \{(\hat{M}_n, \hat{C}_n, \hat{V}_n)\}_{n=1}^N$, $\boldsymbol{Y} = \{(M_n, C_n, V_n)\}_{n=1}^N$
$\hat{S}_k/S_k$	network estimation/training label for the mask of k-*th* cell instance in image I, $\hat{S}_k, S_k \in \{0,1\}^{W \times H}$
$\hat{o}_k/o_k$	network estimation/training label for center coordinate of k-*th* cell instance in the lattice of I
$\mathcal{L}$, $\mathcal{L}_{ce}$, $\mathcal{L}_{l2}$	loss functions used for network optimization

Table S3: The ablation study for the choice of hyper-parameters (optimizer, learning rate, variance σ) in simulation dataset with image and gene IoU scores. The performances corresponding to the chosen parameters are highlighted in bold.

Optimizer	Learning Rate	Dataset	Image IoU					Gene IoU					Dataset	Image IoU					Gene IoU				
			Variance σ					Variance σ						Variance σ					Variance σ				
			3	5	7	9	11	3	5	7	9	11		3	5	7	9	11	3	5	7	9	11
SGD	0.0001	Dense cells High image noise Abundant genes	0.685	0.682	0.731	0.761	0.746	0.734	0.731	0.831	0.854	0.841	Dense cells Low image noise Depleted genes	0.644	0.677	0.758	0.699	0.673	0.766	0.808	0.863	0.824	0.82
	0.001		0.735	0.74	0.755	0.769	0.75	0.817	0.826	0.843	0.858	0.838		0.685	0.741	0.762	0.738	0.700	0.811	0.844	0.868	0.84	0.841
	0.01		0.726	0.728	0.688	0.737	0.724	0.812	0.819	0.77	0.844	0.83		0.668	0.705	0.741	0.719	0.726	0.796	0.828	0.849	0.831	0.85
	0.1		0.703	0.794	0.702	0.687	0.695	0.772	0.764	0.781	0.753	0.778		0.638	0.644	0.661	0.653	0.658	0.759	0.766	0.802	0.781	0.803
Adam	0.0001		0.668	0.721	0.740	0.774	0.688	0.712	0.814	0.836	0.86	0.751		0.653	0.650	0.745	0.733	0.690	0.768	0.779	0.85	0.838	0.829
	0.001		0.712	0.732	0.774	0.783	0.74	0.802	0.821	0.853	0.877	0.836		0.710	0.744	**0.764**	0.752	0.750	0.823	0.847	**0.871**	0.851	0.862
	0.01		0.693	0.707	0.752	0.767	0.764	0.760	0.781	0.841	0.858	0.855		0.688	0.716	0.695	0.674	0.734	0.808	0.833	0.83	0.805	0.854
	0.1		0.665	0.679	0.716	0.706	0.731	0.710	0.727	0.824	0.79	0.832		0.629	0.669	0.656	0.712	0.713	0.754	0.803	0.788	0.829	0.845
AdamW	0.0001		0.731	0.719	0.763	**0.779**	0.737	0.812	0.813	0.85	**0.865**	0.835		0.681	0.658	0.754	0.718	0.699	0.8	0.792	0.858	0.831	0.844
	0.001		0.719	0.753	0.771	0.788	0.755	0.808	0.835	0.851	0.883	0.847		0.708	0.727	0.759	0.761	0.738	0.822	0.837	0.86	0.861	0.856
	0.01		0.679	0.747	0.748	0.733	0.77	0.723	0.83	0.838	0.838	0.854		0.722	0.739	0.774	0.748	0.746	0.828	0.842	0.884	0.844	0.86
	0.1		0.697	0.69	0.714	0.713	0.716	0.763	0.745	0.818	0.824	0.822		0.660	0.692	0.702	0.730	0.721	0.79	0.819	0.834	0.836	0.846

Table S4: The ablation study for the choice of hyper-parameters (optimizer, learning rate, variance σ) in NSCLC dataset with image and gene IoU scores. The performances corresponding to the chosen parameters are highlighted in bold.

Optimizer	Learning Rate	Dataset	Image IoU					Gene IoU				
			Variance σ					Variance σ				
			3	5	7	9	11	3	5	7	9	11
SGD	0.0001	NSCLC	0.688	0.683	0.676	0.692	0.67	0.759	0.752	0.747	0.766	0.74
	0.001		0.708	0.696	0.672	0.702	0.692	0.787	0.766	0.744	0.779	0.765
	0.01		0.673	0.634	0.685	0.714	0.701	0.746	0.699	0.756	0.8	0.781
	0.1		0.659	0.647	0.648	0.676	0.654	0.718	0.71	0.712	0.748	0.716
Adam	0.0001		0.711	0.663	0.697	0.715	0.707	0.797	0.725	0.768	0.8	0.786
	0.001		0.713	0.673	0.708	0.72	0.714	0.798	0.745	0.787	0.804	0.798
	0.01		0.687	0.69	0.657	0.691	0.699	0.757	0.764	0.718	0.764	0.773
	0.1		0.668	0.629	0.633	0.684	0.649	0.727	0.693	0.699	0.756	0.712
AdamW	0.0001		0.721	0.685	0.691	0.728	0.709	0.804	0.755	0.764	0.809	0.787
	0.001		0.719	0.689	0.686	**0.734**	0.711	0.801	0.76	0.756	**0.817**	0.796
	0.01		0.692	0.658	0.668	0.706	0.684	0.765	0.72	0.727	0.785	0.754
	0.1		0.678	0.641	0.664	0.689	0.662	0.75	0.704	0.725	0.761	0.721

Table S5: The ablation study for the choice of hyper-parameters (optimizer, learning rate, variance σ) in the hippocampus dataset. The performances corresponding to the chosen parameters are highlighted in bold.

Optimizer	Learning Rate	Dataset	Variance σ																			
			3					5					7					9				
			Cell Calling			Cell Area (pixel)		Cell Calling			Cell Area (pixel)		Cell Calling			Cell Area (pixel)		Cell Calling			Cell Area (pixel)	
			3 pixel	5 pixel	7 pixel	Avg Area	Avg Radius	3 pixel	5 pixel	7 pixel	Avg Area	Avg Radius	3 pixel	5 pixel	7 pixel	Avg Area	Avg Radius	3 pixel	5 pixel	7 pixel	Avg Area	Avg Radius
SGD	0.0001	Hippocampus	37.93	18.02	10.09	1119.47	18.88	34.33	13.89	7.02	1057.20	18.35	46.30	23.76	15.68	1297.43	20.33	38.26	18.18	10.24	1121.01	18.90
	0.001		39.84	19.58	11.63	1142.15	19.07	36.95	16.91	9.32	1109.14	18.79	47.63	24.20	16.01	1300.94	20.35	40.02	19.92	11.86	1155.11	19.18
	0.01		44.20	21.90	14.80	1199.14	19.54	36.50	16.65	8.99	1103.48	18.75	46.82	24.03	15.70	1298.92	20.34	42.01	21.88	13.05	1168.47	19.29
	0.1		36.89	16.72	9.05	1105.58	18.76	35.19	15.27	7.90	1064.32	18.41	45.37	23.62	15.64	1295.36	20.31	37.74	17.56	9.61	1116.50	18.86
Adam	0.0001		36.37	16.26	8.91	1100.74	18.72	35.56	15.76	8.18	1077.49	18.52	47.18	24.11	15.79	1299.90	20.35	40.38	20.11	12.43	1158.04	19.20
	0.001		42.24	21.90	14.05	1173.87	19.34	38.23	18.27	10.70	1124.86	18.93	47.28	24.13	15.87	1300.24	20.35	44.41	23.52	15.64	1292.87	20.29
	0.01		41.66	20.37	13.43	1160.96	19.23	37.77	17.83	9.95	1117.37	18.86	48.33	24.92	16.02	1311.58	20.44	42.12	22.09	13.78	1172.22	19.32
	0.1		39.17	18.94	11.21	1128.84	18.96	34.85	14.30	7.44	1058.19	18.36	46.67	23.94	15.68	1298.58	20.34	38.87	18.69	10.77	1125.56	18.93
AdamW	0.0001		37.55	17.18	9.40	1110.21	18.80	36.04	16.18	8,76	1083.26	18.57	49.87	25.17	16.91	1352.35	20.75	41.64	20.41	12.88	1160.30	19.22
	0.001		40.45	20.22	12.70	1159.33	19.21	39.68	20.31	13.44	1153.11	19.16	**50.76**	**25.24**	**17.88**	**1364.79**	**20.85**	43.79	23.07	14.92	1188.68	19.46
	0.01		42.75	22.62	14.77	1175.26	19.35	38.88	18.54	11.62	1126.93	18.94	48.62	24.98	16.33	1312.21	20.44	42.81	22.78	14.37	1176.14	19.35
	0.1		38.51	18.47	10.62	1126.03	18.94	35.02	14.96	7.81	1062.27	18.39	48.08	24.36	16.01	1303.32	20.37	39.47	19.21	11.12	1130.29	18.97

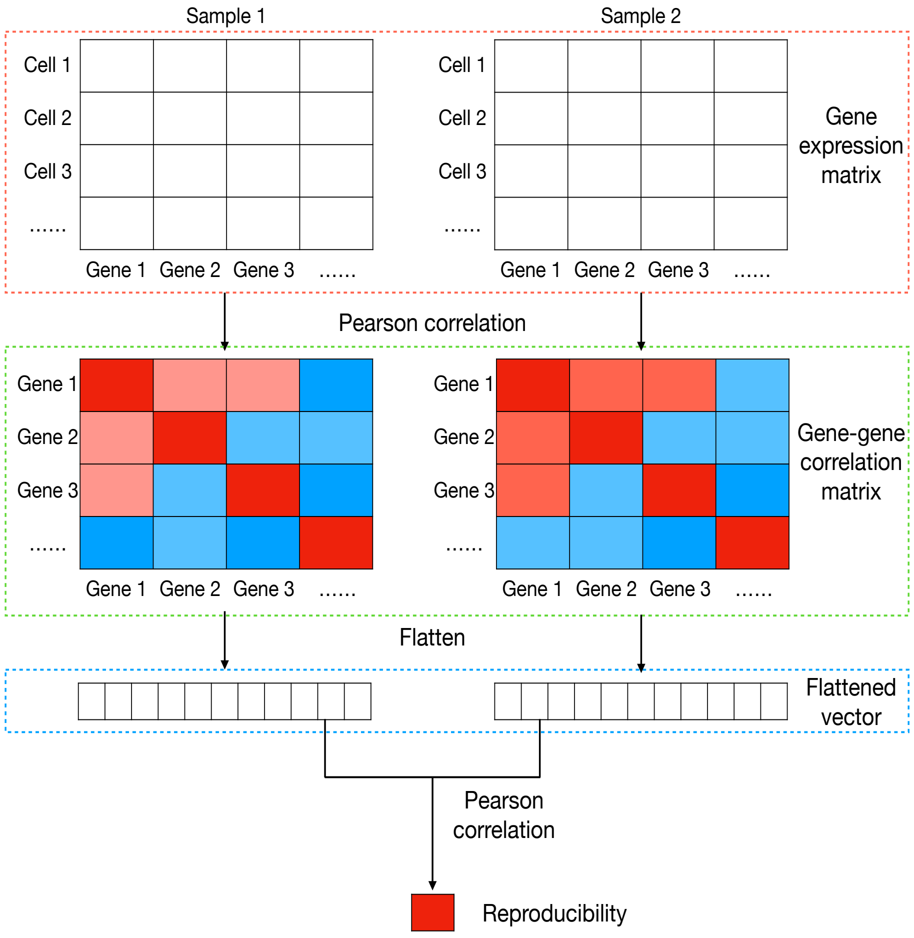

Fig. S3: Overview of the process calculating the reproducibility of gene-gene correlations. Red and blue colors in the matrices indicate positive and negative values, respectively.
