# Supplementary material for: GeneSegNet: a deep learning framework for cell segmentation by integrating gene expression and imaging
Source: Genome Biol. 2023 Oct 19;24:235. doi: 10.1186/s13059-023-03054-0 (PMC10585768; doi:10.1186/s13059-023-03054-0)
Supplement: Supplementary file 3 — Additional file 3. Review history [file 13059_2023_3054_MOESM3_ESM.docx]

**1^st^ round**

**Reviewer 1**

Wang, Wang, and Liu et al. present GeneSegNet, a neural network that takes advantage of both protein images and RNA localization information to perform cell segmentation. The authors describe their novel architecture and training setup, which involves iterative relabeling and training on the noisy input data. They then benchmark their approach against competing methods using a number of custom metrics.

The idea behind their method is interesting, and could potentially increase the accuracy of segmentation. However, in its present form, it's not clear if this is actually the case, and it is challenging to understand what the advantages and disadvantages of this method are compared to competing methods.

Major concerns:

1. The lack of any ground-truth data makes interpreting performance challenging. Since the authors have not generated any of their own ground-truth data, they can't use the previous segmentations to compare against. Instead, the authors have constructed a number of metrics that they use to evaluate performance. However, none of these metrics actually reflect ground truth performance. For example, more transcripts per cells is only a useful metric of performance if those additional transcripts actually belong to that cell. Similarly, larger average cell size is only desirable if it is the correct cell size. The limitations of these metrics are not discussed anywhere, and are instead treated as inherently reliable measures to assess performance.

2. The authors then benchmark their approach against a number of competing methods, using the empirical metrics they defined above. It is clear that GeneSegNet significantly outperforms other methods in Figure 2C with their cell calling score. However, for the rest of Figure 2, GeneSegNet does as well as the other methods, but does not surpass them. There is not a clear differentiation of their method from prior approaches in performance. An increase in performance isn't the only reason that a method could be useful. For example, if a new method is faster, easier to use, or produces more interpretable results, these would all be reasons to select such a method over a competing method, even in the absence of a performance benefit. However, the authors have not commented on any of these potential alternative reasons for selecting their method

3. How do the authors intend their tool to be used? For the analyses presented in the paper, the authors trained their model on a portion of each dataset, and tested on a held-out portion. This gives a reliable estimate of how the model would perform on additional, unseen data from those same datasets, but not on how it would perform on new datasets. Is the pretrained model generalizable enough that we can expect to use it out of the box? Or is the expectation that each user will retrain the model on their own data? The readme in their github guides the user towards training their own model. However, the utility of a segmentation algorithm that needs to be retrained each time is significantly less than one that can be run out of the box, especially since the authors are comparing their method against some algorithms that do not require any dataset-specific training.

Minor concerns
1. The authors have constructed a custom training setup that involves iterative relabeling of the training examples to mitigate the biases in these datasets. However, there is no evaluation of this technique. Is it necessary? How were the hyper-parameters for this new training scheme selected? How sensitive is performance to the selection of these metrics? Right now it is not possible to assess the utility of this approach.
2. We attempted to run the author's code. However, the repo cannot be properly installed as the requirements file has local dependencies which are improperly specified, and hence cannot be installed outside of the author's original compute environment.

**Reviewer 2**

In this very short paper, the authors present GeneSegNet, a segmentation algorithm that utilizes both nuclei channel and RNA spots to perform segmentation. There are several algorithms out there for doing the same type of work (segmenting cells from FISH data for quantifying gene expression). They compared the tool against image-based segmentation and RNA based segmentation algorithms using various metrics such as cell calling and gene-gene correlation across segmented cells. The major concerns regarding this work are as follows:
1. Although the authors used a variety of metrics to compare GeneSegNet against various algorithms the lack of ground truth makes it hard to infer if the method is truly segmenting real cells. We find that the metrics themselves are somewhat flawed since the major selling point of GeneSegNet is that it includes more RNA-spots per cell. However, it is difficult to say whether those included spots really belong to the cell. For instance, there are many cases in tissue RNA imaging that the nuclei of a cell is not detected but the cell body (including RNAs) is. In those cases, I assume that the RNA spots from non-nucleated cells will be erroneously included.

2. The lack of ground truth to really assess segmentation is concerning. 2 experiments should be done:
a. Use a cell membrane marker to delineate cell outlines - compare this segmentation to GeneSegNet.
b. Do a mix cell experiment in that you have RFP vs GFP labelled cytoplasms where you can clearly distinguished which RNAs belong to which cells. then compare this ground truth to GeneSegNet.

3. The CellPose segmentation with both nucleus and cytoplasmic stains in NSCLC data can be considered somewhat of a ground truth as this segmentation is based on multiple cytoplasmic markers. In comparison to that segmentation, GeneSegNet results have much bigger cells that do contain more RNA molecules. This is concerning.

4. It is unclear what the biological significant of the gene-pair correlation. Are the genes used known to be correlated biologically? It is probably untrue that all genes are correlated to each other. It is important that the authors mention which gene pairs they used for this analysis.
Minor:
5. The current framework of GeneSegNet seems to only work on one Image channel - DAPI. It is not clear from the manuscript if this framework also allows for multiple image inputs or if the input can be probability maps for cell segmentation generated from multiple markers.

6. The authors presented a zoomed in view of one segmented cell for comparison but providing a zoomed-out representation of how the entire tissue looks after segmentation is also important for a better comparison for the tissue segmentation across methods.

**Reviewer 3**

Wang et al. developed a new computational method for cell segmentation from imaging based spatial transcriptomic (ST) datasets. The main novelty of this proposed method (called GeneSegNet) is that it integrates both nucleus and RNA distribution information to maintain a balance between these two sources of information. They used a U-net like deep neural network modeling approach and iteratively refines segmentation results by using recursive training. They compared the performance of this method with five state-of-the-art methods and showed their over-perform according to the metrics they selected. Considering the importance and difficulty of cell segmentation in ST data analysis, GeneSetNet offers a new tool which potentially could be widely used by the community. The paper is generally well-written. However, I have a number of concerns as detailed below:

Main Concerns:

1. A claimed advantage of GeneSetNet is that it can segment cell boundaries from DAPI and RNA information alone. This is a bold statement and needs to be rigorously validated. There are datasets that contain cell membrane markers (such as: PMID:36203011). The authors should compare their segmented cell boundary results with the morphology delineated by such markers.

2. In the images shown in the paper, the authors showed individual cells in isolation. In reality every cell is neighboring other cells, and probably the most important challenge in cell segmentation is to delineate the boundary between cells in a crowded area. To evaluate the performance it would be important to visualize the segmentation results in multicellular areas rather than just individual cells.

3. It is clear to me how the cell calling scores are exactly calculated. It assumes any transcript can be classified as either in or out of a cell, but this classification depends on the knowledge of the precise location of cell boundary, which is obviously unknown without cell segmentation. Also, when cells are crowded, the transcripts from neighboring cells can merge into a continuum which makes it difficult to determine which is inside vs out. It is unclear how such a problem can be addressed by the current algorithm.

4. How to identify doublet problem - i.e., transcripts from neighboring cells are assigned to the same cell? This is an important concern in ST data analysis and should be at least discussed in the paper.

5. It is a good idea to use gene correlation as an additional metric for performance evaluation. However, I am surprised to see nearly perfect correlation for the lung data. Could it be overly optimistic? In addition to gene correlation, it may be useful to also consider the impact on downstream analysis such as cell-type annotation and differential gene expression analysis, which have more concrete biological meanings.

6. It is unclear if the method has already been implemented as a software package. As a computational toolbox, the utility will be significantly limited without a user-friendly software package with documentation. In the minimum, the authors should provide codes for reproducing the results in this paper.

7. How much computational resource does it take to run this tool in terms of CPU time and memory? These questions can be important from practical point of view.

**Authors’ response**

Reviewer 1:

Wang, Wang, and Liu et al. present GeneSegNet, a neural network that takes ad- vantage of both protein images and RNA localization information to perform cell segmentation. The authors describe their novel architecture and training setup, which involves iterative relabeling and training on the noisy input data. They then benchmark their approach against competing methods using a number of custom metrics.

The idea behind their method is interesting, and could potentially increase the accuracy of segmentation. However, in its present form, it’s not clear if this is actually the case, and it is challenging to understand what the advantages and disadvantages of this method are compared to competing methods.

We thank the reviewer for the insightful comments and valuable suggestions.

Main concerns:

1. The lack of any ground-truth data makes interpreting performance challenging. Since the authors have not generated any of their own ground-truth data, they can’t use the previous segmentations to compare against. Instead, the authors have constructed a number of metrics that they use to evaluate performance. However, none of these metrics actually reflect ground truth performance. For example, more transcripts per cells is only a useful metric of performance if those additional tran- scripts actually belong to that cell. Similarly, larger average cell size is only desirable if it is the correct cell size. The limitations of these metrics are not discussed any- where, and are instead treated as inherently reliable measures to assess performance.

Response:

We thank the reviewer for the valuable comments. We have added discussions of the limitations of these metrics to the main manuscript:

In addition to evaluations based on ground truth, this study also uses numerous metrics independent of ground truth, such as cell calling, proportion of pixels with high pixel values or with RNA reads that fall within cell boundaries, cell size and elongation, and reproducibility analysis. Note that these metrics cannot fully reflect the actual performance of the methods due to the lack of ground truth and their inherent limitations. For example, the ability to absorb more RNA spots into cell boundaries is only desirable when those RNA spots actually belong to the cells. A larger cell size by a segmentation method is only desirable if it reflects the actual cell size. Although these results serve as a good complement, they need to be interpreted jointly with the evaluations based on ground truth to comprehensively benchmark the performances of different cell segmentation methods.

To address the concern regarding the lack of ground truth data, we introduced a simulation dataset with ground truth cell segmentation and RNA-cell assignments. The simulation study considers four scenarios: 1) a simulation scenario with sparsely distributed cells, abundant amount of RNAs, and high level of image noise; 2) a sim- ulation scenario with sparsely distributed cells, depleted amount of RNAs, and low level of image noise; 3) a simulation scenario with densely distributed cells, abundant amount of RNAs, and high level of image noise; and 4) a simulation scenario with densely distributed cells, depleted amount of RNAs, and low level of image noise. We then quantitatively benchmarked the performance of GeneSegNet and the com- peting methods by comparing their cell segmentation and RNA-gene assignment results with the ground truth using Intersection over Union (IoU) scores. The re- sults, as presented in Figure 3 and Figure 6 in the revised manuscript, demonstrate that Genesegnet provides better segmentation results and has higher IoU scores for both the images and the genes comparing other methods.

Furthermore, we also used the cell segmentation derived from the nucleus and membrane markers in the NSCLC dataset as a ground truth to compare the perfor- mance of different methods. Genesegnet again has higher image and gene IoU scores than other competing methods as shown in Figure 7a-b in the revised manuscript.

2. The authors then benchmark their approach against a number of competing methods, using the empirical metrics they defined above. It is clear that GeneSegNet significantly outperforms other methods in Figure 2C with their cell calling score. However, for the rest of Figure 2, GeneSegNet does as well as the other methods, but does not surpass them. There is not a clear differentiation of their method from prior approaches in performance. An increase in performance isn’t the only reason that a method could be useful. For example, if a new method is faster, easier to use, or produces more interpretable results, these would all be reasons to select such a method over a competing method, even in the absence of a performance benefit. However, the authors have not commented on any of these potential alternative reasons for selecting their method.

Response:

We appreciate the insightful feedback from the reviewer. In our revised manuscript, we use a simulation dataset to clearly demonstrate the advantage of GeneSegNet over other methods. The simulation dataset considers two situations: a situation where the imaging noise is high and there are abundant amount of genes, and a situation where the imaging noise is low and there are depleted amount of genes. Methods that only use imaging information will perform poorly when there is high level of image noise, and methods that only use gene information will perform poorly when there is depleted amount of genes. GeneSegNet, on the other hand, uses both type of information and will not be hugely affected when the image noise is high or when the amount of genes is depleted. For both scenarios, we also sim- ulated two situations where cells are densely or sparsely distributed. Figure 3 and Figure 6 in the revised manuscript show that GeneSegNet outperforms all other competing methods. Specifically, GeneSegNet significantly outperforms Cellpose and watershed, which are methods that only use image information, when there is high level of image noise. GeneSegNet slightly outperforms Cellpose when there is low level of image noise and depleted amount of genes, which is expected since the limited amount of gene information does not provide much additional value in cell segmentation. In all situations, GeneSegNet significantly outperforms Baysor, which only uses gene expression information. We didn’t include JSTA in simulation studies since the current method to simulate spatial data cannot generate distinct cell types, and the cell type information is a required input of JSTA. However, the artificial round shapes of cell segmentation given by JSTA clearly does not match the actual cell boundaries, as demonstrated in Figure 4 and Figure 5.

We have also revised the analysis of the NSCLC dataset and took advantage of the nucleus and membrane markers that are originally available in the dataset. The original NSCLC dataset contains both markers for nucleus and membrane. In the previous manuscript, Cellpose applied to both membrane and nucleus marker was treated as a competing method and we showed that GeneSegNet and Cellpose (nucleus and membrane) are very similar in terms of various metrics we analyzed. In the revised manuscript, we now treat the segmentation results given by Cellpose (nucleus and membrane) as a ground truth that delineates the cell outlines. Figure 7a-b shows the image and gene IoU scores comparing the segmentation given by nucleus and membrane and the segmentation derived by GeneSegNet and other competing methods. GeneSegNet outperforms all competing methods in terms of both image and gene IoU scores. GeneSegNet achieves a larger improvement in gene IoU scores compared to image IoU scores due to its modeling of RNA spatial locations.

In addition, we significantly enhanced the reproducibility analysis of the down- stream analysis in the two real datasets. While some methods have comparable performance as GeneSegNet in some scenarios, they performed poorly in other sce- narios. For example, Cellpose has considerably lower reproducibility score in cell clustering and differential analysis compared to GeneSegNet in the hippocampus dataset (Figure 8h-i). GeneSegNet is the only method that consistently ranks among the best in all scenarios.

We believe that these new results, in addition to the cell calling scores, demon- strate that GeneSegNet has clear advantage over other methods.

In addition to the new figures, we have added the below descriptions to the main manuscript:

Discussions of the new simulation dataset:

We begin by computing the Intersection over Union (IoU) scores for images and genes in the simulation dataset, following protocols in existing studies [22, 23, 24, 8, 10, 25]. The IoU scores measure the similarity of cell boundaries (image IoU, Fig. 6a) and assignments of RNA spots to cells (gene IoU, Fig. 6b) between cell segmentation generated by GeneSegNet or a competing method and the ground truth. Methods with cell segmentation more similar to the gound truth have higher IoU scores. In the four simulation scenarios, GeneSegNet consistently outperforms other methods in both image IoU and gene IoU scores. Cellpose, which is also a deep-learning-based method but does not utilize information of RNA spatial locations, has comparable performance as GeneSegNet when there is low image noise and depleted amount of genes (Fig. 3b,d, Fig. 6a-b), but its performance significantly decreases when the image noise is high (Fig. 3a,c, Fig. 6a-b). In comparison, GeneSegNet still maintains high image and gene IoU scores even when there is limited amount of image or RNA spatial information, demonstrating the advantage of utilizing both types of infor- mation. Watershed and Baysor have far worse performance (Fig. 6a-b), consistent with the observations in Fig. 3. We further studied the performance of GeneSegNet in a crowded region when cells are all neighbouring to each other and the spatial locations of RNA spots form a continuum. The performance of GeneSegNet only decreases slightly when cells are crowded and densely distributed (Fig. 3c-d, Fig. 6a-b) compared to the performance when cells are sparsely distributed (Fig. 3a-b, Fig. 6a-b), showing that GeneSegNet is able to delineate cell boundaries and assign RNAs to cells relatively well in a crowded region.

Discussions of the revised NSCLC dataset:

We next evaluated the performance of cell segmentation methods on the real datasets. Similar to the simulation study, we calculated gene and image IoU scores using the ground truth derived from the nucleus and membrane markers in the NSCLC dataset. GeneSegNet again outperforms other methods and has a signif- icantly higher gene IoU compare to Cellpose (Fig. 7a-b), showing the improved ability of GeneSegNet to correctly assign RNA spots to cells.

Discussions of the revised reproducibility analysis:

Finally, to demonstrate the impact of different cell segmentation methods on down-stream analysis, we performed cell clustering, differential gene analysis, and gene-gene correlation analysis using gene expression matrices created by RNA as- signments to cells (Methods). Cell clustering groups cells with similar gene expres- sion profiles into the same clusters and is useful to identify cell types of individual cells. Differential gene analysis identifies genes with differential gene expression levels across cell clusters that may drive the heterogeneity of cells. Gene-gene cor- relation identifies pathways of genes with similar expression patterns across cells that may share common biological functions, and can be used to construct gene co-expression networks for screening candidate biomarkers or therapeutic targets. Since there is no gold standard in real datasets to directly assess these down-stream analysis, we tested if the results of cell clustering, differential gene analysis, and gene-gene correlations are reproducible across biological replicates (Methods), simi- lar to existing studies [27,28]. GeneSegNet again has the best overall performance in both datasets and for three types of reproducibility (Fig. 7j-l and Fig. 8h-j). Other competing methods perform poorly in at least one scenario. For example, Cellpose has significantly lower reproducibility compared to GeneSegNet for cell clustering and differntial gene analysis in the hippocampus dataset (Fig. 8h-i). These results demonstrate that down-stream analyses with GeneSegNet are more stable and re- producible.

3. How do the authors intend their tool to be used? For the analyses presented in the paper, the authors trained their model on a portion of each dataset, and tested on a held-out portion. This gives a reliable estimate of how the model would perform on additional, unseen data from those same datasets, but not on how it would perform on new datasets. Is the pretrained model generalizable enough that we can expect to use it out of the box? Or is the expectation that each user will retrain the model on their own data? The readme in their github guides the user towards training their own model. However, the utility of a segmentation algorithm that needs to be retrained each time is significantly less than one that can be run out of the box, especially since the authors are comparing their method against some algorithms that do not require any dataset-specific training.

Response:

Thank you for your valuable comments. We expect that GeneSegNet can be used by either retraining a new model on a portion of the new data or applying a pre- trained model to the new data. To test the generalizability of a pretrained model, we conducted the following experiments:

1. Train GeneSegNet on the hippocampus dataset and apply the trained model to the NSCLC dataset. 2. Train GeneSegNet on the NSCLC dataset and apply the trained model to the two simulation datasets.

3. Train GeneSegNet on the hippocampus dataset and apply the trained model to the two simulation datasets. As a comparison, we conducted similar experiments for Cellpose, since Cellpose also uses a deep-learning-based framework. In addition, we also applied a pre-trained model of Cellpose downloaded from Cellpose website to the NSCLS dataset and two simulation datasets.

The image IoU and gene IoU of these generalizability experiments, as well as the results of the previous analysis where GeneSegNet and Cellpose were trained and tested on the same dataset, are shown in Supplementary Figure S1. For GeneSegNet, the IoU scores of the generalizability experiments only decrease slightly compared to the IoU scores when GeneSegNet was trained and tested on the same dataset, showing that GeneSegNet can be generalized to new datasets when trained on a different dataset. In comparison, the IoU scores of Cellpose generalizability experi- ments drop considerably in some situations, especially when the Cellpose pre-trained model is used. Thus, GeneSegNet shows comparable or even higher generalizability than Cellpose, and users can readily apply the pre-trained models to new datasets with similar segmentation performance.

The instructions of how to use the pre-trained model was already included in the github page of the software.

We have included the new Supplementary Figure S1 and the following discussions in the main manuscript:

In this study, GeneSegNet and Cellpose were trained and tested on the same datasets with different data splits for evaluation purpose. In real practice, cell seg- mentation methods will be more user-friendly if a model pre-trained on one dataset can be directly applied to another new dataset without additional training. We further tested the performance of GeneSegNet and Cellpose when they are trained on one dataset and tested on a different dataset, and the gene and image IoU scores of GeneSegNet only decrease slightly (Supplementary Fig. 1). The degree of performance loss is slightly less than that of Cellpose, showing that GeneSegNet models have higher generalizability. Thus, a pre-trained model by GeneSegNet can be readily applied to a new dataset.

Minor concerns:

1. The authors have constructed a custom training setup that involves iterative re- labeling of the training examples to mitigate the biases in these datasets. However, there is no evaluation of this technique. Is it necessary? How were the hyper- parameters for this new training scheme selected? How sensitive is performance to the selection of these metrics? Right now it is not possible to assess the utility of this approach.

Response:

We thank the reviewer for valuable comments. We have now added an evaluation of this custom training step by calculating the image and gene IoU scores in the simulation datasets if GeneSegNet stops at the first, second, and third recursive training rounds. Note that GeneSegNet with only the first training round represents a situation where the custom training step is not included. Figure 6c-d shows the results. In all simulation scenarios, both image and gene IoU increases with the number of recursive training rounds, and using recursive training leads to 5% to 15% performance gain compared to not using recursive training. These results indicate that the custom training step contributes to an increase in performance and is thus useful in GeneSegNet framework.

By default, the iterative training process stops after three iterations. We set this stoppling rule for three reasons. First, in real practice we observe that the perfor- mance almost converges at the third iteration and running additional iterations may not lead to a significant increase in performance (Figure 6c-d). Second, since Gene- SegNet is based on a deep-learning framework which is computationally intensive, running additional iterations may significantly increase running time, which will affect the usability of GeneSegNet especially when it is applied to large datasets. Third, since we are already choosing optimal values for other parameters (discussed below), it is not computationally feasible to further explore the optimal choice of the stopping rule. However, users still have the freedom to set the number of iterative training rounds in real applications.

GeneSegNet does employ a process to select the optimal hyperparameters used for training. GeneSegNet was performed with different choices of learning rate (0.01, 0.001, and 0.0001), optimizer (Adam and AdamW), and variance σ (5, 7, and 9), and the set of parameters that leads to the highest cell calling score (3 pixels) was chosen. In an ablation study newly added to the revised manuscript, we show that the performance of GeneSegNet is not hugely affected by different choices of parameters (Supplementary Table S3-S5). Thus, we conclude that the performance of GeneSegNet is not sensitive to the selection of hyperparameters.

We have added the below descriptions in the main manuscript:

Discussions of how performance increases with recursive training rounds:

To evaluate how the recursive training strategy employed by GeneSegNet con- tributes to improved cell segmentation, we also compared the gene and image IoU scores across the first three training iterations of GeneSegNet (Fig.6c-d). Note that GeneSegNet with only the first training iteration is equivalent to the situation where the recursive training strategy is not applied. In all simulation scenarios, both im- age and gene IoU scores increase with the number of recursive training iterations, and using the recursive training strategy leads to 5% to 15% performance gains compared to not doing so. Moreover, the performance of the second and third it- erations are highly similar, showing that the recursive training converges quickly and running three iterations is enough. Thus, GeneSegNet runs for three recursive training iterations by default.

Discussions of the new ablation study:

In this study, GeneSegNet was performed with different choices of learning rate (0.01, 0.001, and 0.0001), optimizer (Adam and AdamW), and variance σ (5, 7, and 9), and the set of parameters that leads to the highest cell calling score (3 pixels) was chosen. In an ablation study, we show that the performance of GeneSegNet is not hugely affected by different choices of parameters (Supplementary Table S3-S5).

2. We attempted to run the author’s code. However, the repo cannot be prop- erly installed as the requirements file has local dependencies which are improperly specified, and hence cannot be installed outside of the author’s original compute environment.

Response:

Thank you for your valuable comments. We removed the local dependencies and updated the requirements file on GitHub. To test the usability of the software, we were able to properly install the software on a new computer. For your conve- nience, we also uploaded our software repository to Code Ocean with a configured environment so that the code can be directly run according to the guidance. The Code Ocean link is: https://codeocean.com/capsule/9320020/tree/v2 , which is also provided in the manuscript data available section.

Reviewer 2:

In this very short paper, the authors present GeneSegNet, a segmentation algorithm that utilizes both nuclei channel and RNA spots to perform segmentation. There are several algorithms out there for doing the same type of work (segmenting cells from FISH data for quantifying gene expression). They compared the tool against image-based segmentation and RNA based segmentation algorithms using various metrics such as cell calling and gene-gene correlation across segmented cells. The major concerns regarding this work are as follows:

We thank the reviewer for the insightful comments and valuable suggestions.

1. Although the authors used a variety of metrics to compare GeneSegNet against various algorithms the lack of ground truth makes it hard to infer if the method is truly segmenting real cells. We find that the metrics themselves are somewhat flawed since the major selling point of GeneSegNet is that it includes more RNA- spots per cell. However, it is difficult to say whether those included spots really belong to the cell. For instance, there are many cases in tissue RNA imaging that the nuclei of a cell is not detected but the cell body (including RNAs) is. In those cases, I assume that the RNA spots from non-nucleated cells will be erroneously included.

Response:

We appreciate the valuable feedback provided. We agree with the reviewer that the metrics used in the previous version of the manuscript are somewhat flawed as there is no ground truth whether the RNA spots really belong to cells. In response to the concerns raised regarding the lack of ground truth data and the accuracy of gene inclusions, we have taken significant measures to address these issues.

First, we have introduced a comprehensive simulation dataset that includes ground truth for both cell boundaries and assignments of RNAs to cells. This addresses the issue that the ground truth of RNA-cell assignment is lacking and facilitates a direct and rigorous comparison between the predictions made by Gene- SegNet and competing models. The simulation dataset considers two situations: a situation where the imaging noise is high and there are abundant amount of genes, and a situation where the imaging noise is low and there are depleted amount of genes. Methods that only use imaging information will perform poorly when there is high level of image noise, and methods that only use gene information will per- form poorly when there is depleted amount of genes. GeneSegNet, on the other hand, uses both type of information and will not be hugely affected when the im- age noise is high or when the amount of genes is depleted. For both scenarios, we also simulated two situations where cells are densely or sparsely distributed. Figure 3 and Figure 6 in the revised manuscript show that GeneSegNet outperforms all other competing methods. Specifically, GeneSegNet significantly outperforms Cell- pose and watershed, which are methods that only use image information, when there is high level of image noise. GeneSegNet slightly outperforms Cellpose when there is low level of image noise and depleted amount of genes, which is expected since the limited amount of gene information does not provide much additional value in cell segmentation. In all situations, GeneSegNet significantly outperforms Baysor, which only uses gene expression information. We didn’t include JSTA in simulation studies since the current method to simulate spatial data cannot generate distinct cell types, and the cell type information is a required input of JSTA. However, the artificial round shape of cell segmentation given by JSTA clearly does not match the actual cell boundaries, as demonstrated in Figure 4 and Figure 5.

Second, we revised the analysis for NSCLC dataset by using the cell segmentation derived from the nucleus and membrane markers as a ground truth to compare the performance of different methods, which will be discussed in detail in below responses. Using the nucleus and membrane markers as a ground truth largely resolves the issue of non-nucleated RNA spots and is a good supplement to the simulation studies. The results are included in Figure 7a-b in the revised manuscript and the segmentation by GeneSegNet has the best agreement with the segmentation derived from the nucleus and membrane markers.

In summary, we believe the new simulation dataset and the revised NSCLC anal- ysis have largely resolved the issue that a ground truth of cell boundary is lacking and supports our claim that GeneSegNet better assigns RNA spots to cells.

In the revised manuscript, we have also added the below descriptions to the main manuscript in addition to the new figures:

Discussions of the new simulation dataset:

We begin by computing the Intersection over Union (IoU) scores for images and genes in the simulation dataset, following protocols in existing studies [22, 23, 24, 8, 10, 25]. The IoU scores measure the similarity of cell boundaries (image IoU, Fig. 6a) and assignments of RNA spots to cells (gene IoU, Fig. 6b) between cell segmentation generated by GeneSegNet or a competing method and the ground truth. Methods with cell segmentation more similar to the gound truth have higher IoU scores. In the four simulation scenarios, GeneSegNet consistently outperforms other methods in both image IoU and gene IoU scores. Cellpose, which is also a deep-learning-based method but does not utilize information of RNA spatial locations, has comparable performance as GeneSegNet when there is low image noise and depleted amount of genes (Fig. 3b,d, Fig. 6a-b), but its performance significantly decreases when the image noise is high (Fig. 3a,c, Fig. 6a-b). In comparison, GeneSegNet still maintains high image and gene IoU scores even when there is limited amount of image or RNA spatial information, demonstrating the advantage of utilizing both types of infor- mation. Watershed and Baysor have far worse performance (Fig. 6a-b), consistent with the observations in Fig. 3. We further studied the performance of GeneSegNet in a crowded region when cells are all neighbouring to each other and the spatial locations of RNA spots form a continuum. The performance of GeneSegNet only decreases slightly when cells are crowded and densely distributed (Fig. 3c-d, Fig. 6a-b) compared to the performance when cells are sparsely distributed (Fig. 3a-b, Fig. 6a-b), showing that GeneSegNet is able to delineate cell boundaries and assign RNAs to cells relatively well in a crowded region.

Discussions of the revised NSCLC dataset:

We next evaluated the performance of cell segmentation methods on the real datasets. Similar to the simulation study, we calculated gene and image IoU scores using the ground truth derived from the nucleus and membrane markers in the NSCLC dataset. GeneSegNet again outperforms other methods and has a signifi- cantly higher gene IoU compare to Cellpose(Fig. 7a-b), showing the improved ability of GeneSegNet to correctly assign RNA spots to cells.

2. The lack of ground truth to really assess segmentation is concerning. 2 experi- ments should be done:

a.Use a cell membrane marker to delineate cell outlines -– compare this segmenta- tion to GeneSegNet.

Response:

We appreciate this very useful suggestion. We have revised the analysis of the NSCLC dataset and took advantage of the nucleus and membrane markers that are originally available in the dataset. The original NSCLC dataset contains both markers for nucleus and membrane. In the previous manuscript, Cellpose applied to both membrane and nucleus marker was treated as a competing method and we showed that GeneSegNet and Cellpose (nucleus and membrane) are very similar in terms of various metrics we analyzed. In the revised manuscript, we now treat the segmentation results given by Cellpose (nucleus and membrane) as a ground truth that delineates the cell outlines.

Figure 7a-b shows the image and gene IoU scores comparing the segmentation given by nucleus and membrane and the segmentation generated by GeneSegNet and other competing methods. GeneSegNet outperforms all competing methods in terms of both image and gene IoU. GeneSegNet achieves a larger improvement in gene IoU compared to image IoU due to its modeling of RNA spatial locations.

We have added the new Figure 7a-b and the following discussions in the main manuscript:

We next evaluated the performance of cell segmentation methods on the real datasets. Similar to the simulation study, we calculated gene and image IoU scores using the ground truth derived from the nucleus and membrane markers in the NSCLC dataset. GeneSegNet again outperforms other methods and has a signifi- cantly higher gene IoU compare to Cellpose(Fig. 7a-b), showing the improved ability of GeneSegNet to correctly assign RNA spots to cells. b.Do a mix cell experiment in that you have RFP vs GFP labelled cytoplasms where you can clearly distinguished which RNAs belong to which cells. then com- pare this ground truth to GeneSegNet.

Response:

We appreciate this insightful suggestion. We have searched the literature but unfortunately were not able to identify a mix cell experiment that has RFP vs GFP labelled cytoplasms and also has in situ measurements of RNA spatial locations. To overcome this issue, we designed a simulation experiment where we simulated ground truth of both cell segmentation and RNA-cell assignments (which RNAs belong to which cells). The details of the simulation study has already been discussed in the previous response to the first comment.

In addition, we argue that using the nucleus and membrane markers provided in NSCLC are already enough to clearly distinguish which RNAs belong to which cells in the NSCLC dataset. Although the membrane markers in NSCLC dataset do not have two colors (e.g., RFP vs GFP), a single RNA can still be unambiguously assigned to a cell if the spatial location of the RNA is within the cell boundary of that cell. This is because the cell boundaries are non-overlapping and the spatial location of a single RNA is unique, so the RNA can be assigned to at most one cell. In this case, the color information of RFP and GFP may not provide additional information to improve the assignment of an RNA to a cell.

3. The CellPose segmentation with both nucleus and cytoplasmic stains in NSCLC data can be considered somewhat of a ground truth as this segmentation is based on multiple cytoplasmic markers. In comparison to that segmentation, GeneSegNet results have much bigger cells that do contain more RNA molecules. This is con- cerning.

Response:

Thank you for the insightful feedback. As discussed above, we have revised the analysis of the NSCLC dataset and considered the Cellpose segmentation with both nucleus and membrane stains as the ground truth.

We would like to respectively argue that the GeneSegNet results do not yield much bigger cells compared to the Cellpose segmentation with both nucleus and membrane stains for three reasons. First, in Figure 2g of the previous version of the manuscript, we showed that the distributions of cell size generated by GeneSegNet and Cellpose (nucleus and membrane) are very similar and almost have the same median values. Second, we quantitatively calculated the image IoU score between segmentations generated by GeneSegNet and Cellpose (nucleus and membrane), which is shown as Figure 7a in the revised manuscript. The image IoU score reflects how similar the two segmentations are. GeneSegNet achieves an IoU score of 0.734, is slightly higher than Cellpose that only uses nucleus, and is much higher than other competing methods. Thus the cell size by GeneSegNet cannot be much larger than Cellpose (nucleus and membrane), since otherwise the image IoU score will be very low. Third, examples of cells in Figure 4 also show that segmentations given by GeneSegNet is highly similar to the ground truth, and the size of the cell inferred by GeneSegNet is close to that by the ground truth.

4. It is unclear what the biological significant of the gene-pair correlation. Are the genes used known to be correlated biologically? It is probably untrue that all genes are correlated to each other. It is important that the authors mention which gene pairs they used for this analysis.

Response:

We thank the reviewer for the important comment. We apologize that in the previous manuscript, the x-axis label of Figure 2j is misleading. The axis label should be ’Reproducibility’ instead of ’Correlation’. We have fixed the x-axis label in the revised manuscript (which is now Figure 7l and Figure 8j).

Gene-gene correlation is an important analysis for genomic datasets with gene expression measurements. It can be used to identify groups of genes that are co- expressed and may share the same biological functions and to derive gene co- expression networks to understand the molecular mechanisms of a biological system. The gene-gene correlation analysis is a basis of many well-known computational methods for analyzing genomic datasets, such as WGCNA (Langfelder et al. 2008, PMID: 19114008, 15086 Google Scholar citations).

The reviewer is totally correct that not all genes are correlated biologically. What we are showing is the reproducibility of the gene-gene correlation calculated on biological replicates using the gene expression values obtained by different segmen- tation methods. A high reproducibility score means that the gene-gene correlation calculated from different biological replicates are highly similar and reproducible, but the actual gene-gene correlations can be both positive and negative. For ex- ample, if the gene-gene correlation is calculated as -0.5 in one biological replicate and -0.52 in another biological replicate, they are highly reproducible and will con- tribute to a high reproducibility score. We evaluate the reproducibility instead of the gene-gene correlation itself since there is no ground truth of the gene-gene cor- relation to be compared to. The reproducibility analysis strategy is also used in other published studies, such as Junttila et al. 2022 (PMID: 35880426) and Kang et al. 2021 (PMID: 33828580).

We have provided the list of genes used in the analysis as Supplementary Table S6. We have also added the below descriptions in the main manuscript to discuss the biological significance of the gene-gene correlation analysis, along with other downstream analyses:

Cell clustering groups cells with similar gene expression profiles into the same clusters and is useful to identify cell types of individual cells. Differential gene anal- ysis identifies genes with differential gene expression levels across cell clusters that may drive the heterogeneity of cells. Gene-gene correlation identifies pathways of genes with similar expression patterns across cells that may share common biological functions, and can be used to construct gene co-expression networks for screening candidate biomarkers or therapeutic targets [25].

5. The current framework of GeneSegNet seems to only work on one Image chan- nel - DAPI. It is not clear from the manuscript if this framework also allows for multiple image inputs or if the input can be probability maps for cell segmentation generated from multiple markers.

Response:

We thank the reviewer for the valuable comments. GeneSegNet now supports multiple image channels as input. We added the explanation about how to change the number of channels as input in the README.md file of GitHub (See parame- ter "chan" in “Training from scratch” section). Although not tested in this study, GeneSegNet in principle can also accept probability maps for cell segmentation as input, as long as the probability maps are of the same format as an ordinary image. We have added the below discussions in the main manuscript:

GeneSegNet supports multiple image channels as input, and also accepts proba- bility maps for cell segmentation as input, as long as the probability maps have the same data format as ordinary images.

6. The authors presented a zoomed in view of one segmented cell for comparison but providing a zoomed-out representation of how the entire tissue looks after seg- mentation is also important for a better comparison for the tissue segmentation across methods.

Response:

We thank the reviewer for the valuable comments. We agree with the reviewer that a zommed-out representation is important to compare across tissue segmentation methods. We have experimented with visualizing the entire tissue but it is not possible to clearly see the location of a cell, the location of individual RNA spots, and the cell segmentation boundaries, and it is visually hard to compare the performance across different methods. Thus, we have tried to include as many cells as possible in the zoomed-out view while it is still possible to see individual cells, RNA spots, and cell segmentation boundaries. We have added the zoomed-out representation for the new simulation study in Figure 3, for the NSCLC dataset in Figure 4b, and for the hippocampus dataset in Figure 5b.

Reviewer 3:

Wang et al. developed a new computational method for cell segmentation from imag- ing based spatial transcriptomic (ST) datasets. The main novelty of this proposed method (called GeneSegNet) is that it integrates both nucleus and RNA distribution information to maintain a balance between these two sources of information. They used a U-net like deep neural network modeling approach and iteratively refines segmentation results by using recursive training. They compared the performance of this method with five state-of-the-art methods and showed their over-perform according to the metrics they selected. Considering the importance and difficulty of cell segmentation in ST data analysis, GeneSetNet offers a new tool which poten- tially could be widely used by the community. The paper is generally well-written. However, I have a number of concerns as detailed below:

We thank the reviewer for the insightful comments and valuable suggestions.

Main Concerns:

1. A claimed advantage of GeneSetNet is that it can segment cell boundaries from DAPI and RNA information alone. This is a bold statement and needs to be rig- orously validated. There are datasets that contain cell membrane markers (such as: PMID:36203011). The authors should compare their segmented cell boundary results with the morphology delineated by such markers.

Response:

We appreciate this very useful suggestion. In response to your suggestions, we have revised the analysis of the NSCLC dataset and took advantage of the nucleus and membrane markers that are originally available in the dataset. The NSCLC dataset used in this study is the same study suggest by the reviewer (i.e., PMID:36203011).

The original NSCLC dataset contains both markers for nucleus and membrane. In the previous manuscript, Cellpose applied to both membrane and nucleus marker was treated as a competing method and we showed that GeneSegNet and Cellpose (nucleus and membrane) are very similar in terms of various metrics we analyzed. In the revised manuscript, we now treat the segmentation results given by Cellpose (nucleus and membrane) as a ground truth that delineates the cell outlines. Figure 7a-b shows the image and gene IoU scores comparing the segmentation given by nucleus and membrane and the segmentation generated by GeneSegNet and other competing methods. GeneSegNet outperforms all competing methods in terms of both image and gene IoU. GeneSegNet achieves a larger improvement in gene IoU compared to image IoU due to its modeling of RNA spatial locations. We have added the new Figure 7a-b, revised other parts of Figure 4 and Figure 7 to reflect the change that segmentation by nucleus and membrane markers is now used as ground truth. We have also added below descriptions in the revised manuscript:

We next evaluated the performance of cell segmentation methods on the real datasets. Similar to the simulation study, we calculated gene and image IoU scores using the ground truth derived from the nucleus and membrane markers in the NSCLC dataset. GeneSegNet again outperforms other methods and has a signifi- cantly higher gene IoU compare to Cellpose(Fig. 7a-b), showing the improved ability of GeneSegNet to correctly assign RNA spots to cells.

2. In the images shown in the paper, the authors showed individual cells in isolation. In reality every cell is neighboring other cells, and probably the most important chal- lenge in cell segmentation is to delineate the boundary between cells in a crowded area. To evaluate the performance it would be important to visualize the segmen- tation results in multicellular areas rather than just individual cells.

Response:

We thank the reviewer for the valuable comment. We have now added visualization of segmentation in multi-cellular areas in addition to individual cells. The new figures are Figure 3 for the new simulation studies, Figure 4b for the NSCLC dataset, and Figure 5b for the hippocampus dataset. The results still show that GeneSegNet outperforms other methods in terms of cell segmentation and assigning RNA spots to cells in most cases.

3. It is clear to me how the cell calling scores are exactly calculated. It assumes any transcript can be classified as either in or out of a cell, but this classification de- pends on the knowledge of the precise location of cell boundary, which is obviously unknown without cell segmentation. Also, when cells are crowded, the transcripts from neighboring cells can merge into a continuum which makes it difficult to de- termine which is inside vs out. It is unclear how such a problem can be addressed by the current algorithm.

Response:

We thank the reviewer for the insightful comment. We would like to first clarify that the current cell calling algorithm depends on the cell boundary generated by a cell segmentation method, but we don’t assume that the cell boundary has to be precise. The idea is that a good cell segmentation will include most RNAs within the cell boundary, and the RNAs directly outside the cell boundary should be relatively sparse. This is because we assume that most RNAs stay within the cell boundaries. We fully agree with the reviewer that the cell calling algorithm may encounter problems when cells are crowded and there is a continuum of RNA transcripts. When we first developed the cell calling algorithm, the cell calling score was calcu- lated for each cell individually, and then all cell calling scores were averaged as an overall score. Thus, the assumption that RNAs directly outside the cell boundary is relatively sparse may fail, since there could be RNAs from other cells that are within a small neighborhood outside the cell boundary. So we revised the cell calling algorithm. The current algorithm we use in this version and previous version of the manuscript determines the whole set of RNAs that reside in any of the cell, and the whole set of RNAs that reside in a close neighborhood of any of the cell. The cell calling score is then defined as the proportion of the cardinalities of the two sets. In this way, the problem of crowded cells is largely resolved, since multiple crowded cells will be essentially treated as a whole, and there is no need to identify if one RNA spot belongs to a certain cell.

Nevertheless, cell calling is still a relatively indirect measurement. Thus, we also used ground truth to directly evaluate the cell segmentation performance of dif- ferent methods. First, we introduced a simulation dataset with ground truth cell segmentation and RNA-cell assignments. This allows us to precisely evaluate if the cell segmentation generated by a method can accurately assign an RNA to its cor- responding cell. The simulation study considers four scenarios: 1) a simulation sce- nario with sparsely distributed cells, abundant RNAs within cells, and high level of image noise; 2) a simulation scenario with sparsely distributed cells, depleted RNAs within cells, and low level of image noise; 3) a simulation scenario with densely dis- tributed cells, abundant RNAs within cells, and high level of image noise; and 4) a simulation scenario with densely distributed cells, depleted RNAs within cells, and low level of image noise. We then quantitatively benchmarked the performance of GeneSegNet and the competing methods by comparing their cell segmentation and RNA-gene assignment results with the ground truth using Intersection over Union (IoU) scores. The results, as presented in Figure 3 and Figure 6 in the revised manuscript, demonstrate that Genesegnet provides better segmentation results and has higher IoU scores for both the images and the genes comparing other methods. Second, as discussed in the previous response, we utilized the cell membrane markers in the NSCLC dataset to serve as a ground truth of cell segmentation. By calculating image and gene IoU scores, we were able to directly compare the segmentation generated by a method with the ground truth. The results again show that GeneSegNet has the best performance.

We have added the following discussions about the cell calling algorithm in the main manuscript:

Compared to calculating cell calling scores separately within each cell and averag- ing cell calling scores across cells, the current approach is able to handle a situation where cells are crowded, RNAs from neighboring cells merge into a continuum, and differentiating RNAs within or outside cells is difficult.

4. How to identify doublet problem - i.e., transcripts from neighboring cells are assigned to the same cell? This is an important concern in ST data analysis and should be at least discussed in the paper.

Response:

We thank the reviewer for this valuable comment. We agree with the reviewer that the doublet problem is an important challenge in analyzing ST data. In the revised manuscript, the simulation study was designed in a way that cells are either sparsely or densely distributed. Since we know the ground truth of cell segmentation and RNA-cell assignment in the simulation, the dense cell situation allows us to evaluate the performance of GeneSegNet when there are lots of doublets. Figure 3c-d shows that the simulation scenarios with densely distributed cells are able to mimic a situation where cells are crowded and transcripts from neighboring cells are merged. Compared to simulation scenarios where cells are sparsely distributed, the image and gene IoU of GeneSegNet does drop when cells are densely distributed, but the level of decrease is relatively small. When there is low image noise and depleted genes, the image IoU drops from 0.788 in sparse cell situation to 0.764 in dense cell situation, and the gene IoU drops from 0.926 in sparse cell situation to 0.871 in dense cell situation. When there is high image noise and abundant genes, the image IoU drops from 0.824 in sparse cell situation to 0.779 in dense cell situation, and the gene IoU drops from 0.977 in sparse cell situation to 0.865 in dense cell situation. In addition, GeneSegNet still outperforms other methods in the dense cell scenarios. These results show that GeneSegNet is not severely impacted by the crowded cell situation and can still generate reliable cell segmentation results that outperforms other methods.

In addition, as discussed in the previous response, we visualized the multi-cellular segmentation of different methods in Figure 3c-d, Figure 4b, and Figure 5b. These results show that GeneSegNet is still able to reliably provide cell segmentation even when cells are croweded.

We have included the below discussion in the main manuscript

We further studied the performance of GeneSegNet in a crowded region when cells are all neighbouring to each other and the spatial locations of RNA spots form a continuum. The performance of GeneSegNet only decreases slightly when cells are crowded and densely distributed (Fig. 3c-d, Fig. 6a-b) compared to the performance when cells are sparsely distributed (Fig. 3a-b, Fig. 6a-b), showing that GeneSegNet is able to delineate cell boundaries and assign RNAs to cells relatively well in a crowded region.

5. It is a good idea to use gene correlation as an additional metric for performance evaluation. However, I am surprised to see nearly perfect correlation for the lung data. Could it be overly optimistic? In addition to gene correlation, it may be useful to also consider the impact on downstream analysis such as cell-type annotation and differential gene expression analysis, which have more concrete biological meanings.

Response:

We thank the reviewer for the insightful comments. We apologize that in the previous manuscript, the x-axis label of Figure 2j is misleading. The axis label should be ’Reproducibility’ instead of ’Correlation’. We have fixed the x-axis label in the revised manuscript (which is now Figure 7l and Figure 8j). The reviewer is totally correct that genes are not perfectly correlated. What we are showing is the reproducibility of the gene-gene correlation calculated on biolog- ical replicates using the gene expression values obtained by different segmentation methods. A high reproducibility score means that the gene-gene correlation calcu- lated from different biological replicates are highly similar and reproducible, but the actual gene-gene correlations can be both positive and negative. For example, if the gene-gene correlation is calculated as -0.5 in one biological replicate and -0.52 in another biological replicate, they are highly reproducible and will contribute to a high reproducibility score. We evaluate the reproducibility instead of the gene-gene correlation itself since there is no ground truth of the gene-gene correlation to be compared to. The reproducibility analysis strategy is also used in other published studies, such as Junttila et al. 2022 (PMID: 35880426) and Kang et al. 2021 (PMID: 33828580).

In the revised manuscript, we also included reproducibility analysis for cell cluster- ing and differential gene expression analysis, for the same reason that a ground truth is lacking for directly evaluating the performances. We did not perform the analy- sis for cell-type annotation since cell-type annotation is usually done by manually annotating cell types using top differential genes, making it hard to objectively eval- uate the performance. Plus, since the cell type annotation depends on cell clustering and the differential analysis, we argue that a good performance in cell clustering and differential analysis should also lead to a reasonably good cell-type annotation. The revised reproducibility analysis are now included in Figure 7j-l and Figure 8h- j. The results show that GeneSegNet has the best overall performance for all the reproducibility analysis.

We have added the below descriptions in the main manuscript:

Finally, to demonstrate the impact of different cell segmentation methods on down-stream analysis, we performed cell clustering, differential gene analysis, and gene-gene correlation analysis using gene expression matrices created by RNA as- signments to cells (Methods). Cell clustering groups cells with similar gene expres- sion profiles into the same clusters and is useful to identify cell types of individual cells. Differential gene analysis identifies genes with differential gene expression levels across cell clusters that may drive the heterogeneity of cells. Gene-gene cor- relation identifies pathways of genes with similar expression patterns across cells that may share common biological functions, and can be used to construct gene co-expression networks for screening candidate biomarkers or therapeutic targets.

Since there is no gold standard in real datasets to directly assess these down-stream analysis, we tested if the results of cell clustering, differential gene analysis, and gene-gene correlations are reproducible across biological replicates (Methods), sim- ilar to existing studies [27,28]. GeneSegNet again has the best overall performance in both datasets and for three types of reproducibility (Fig. 7j-l and Fig. 8h-j), showing that the down-stream analysis results with GeneSegNet are more stable and reproducible.

6. It is unclear if the method has already been implemented as a software package. As a computational toolbox, the utility will be significantly limited without a user- friendly software package with documentation. In the minimum, the authors should provide codes for reproducing the results in this paper. Add clarification.

Response:

We thank the reviewer for the valuable comment. We have uploaded the code to GitHub with a detailed manual page of installing and using the software. The URL to the GitHub repository is https://github.com/BoomStarcuc/GeneSegNet.

We have also uploaded our repository to the Code Ocean with a configured envi- ronment. The code can be directly executed according to the guidance. The link is:

https://codeocean.com/capsule/9320020/tree/v2

We have added below descritpions in the main manuscript:

GeneSegNet software package is available on GitHub: https://github.com/BoomStarcuc/GeneSegNet, on Code Ocean: https://codeocean.com/capsule/9320020/tree/v2 with a config- ured environment, or on Zenodo https://zenodo.org/record/8102672 with DOI 10.5281/zenodo.8102672.

7. How much computational resource does it take to run this tool in terms of CPU time and memory? These questions can be important from practical point of view.

Response:

We thank the reviewer for this valuable comment. GeneSegNet uses GPU for training a cell segmentation model and CPU for making inference using the trained model. We have listed the computational time, memory usage, and configurations in Supplementary Table S2 for running all datasets used in this study (simulation studies, NSCLC dataset, and hippocampus dataset). Using 8GB GPU memory, GeneSegNet can finish running within a day for all datasets in this study. We expect that the running time of GeneSegNet will be further decreased if users have access to more powerful GPU resources.

We have also added the below discussion in the main manuscript:

Although GeneSegNet’s recursive training strategy will lead to increased running time, GeneSegNet is still able to finish running within 24 hours for all the datasets with 8GB GPU memory (Supplementary Table S2). We expect the running time can be further decreased if a more powerful GPU is available.

**2^nd^ round**

**Reviewer 1**

The authors have addressed all of our previous comments.

**Reviewer 2**

The authors presented a revised manuscript of the paper “GeneSegNet” that performs cell segmentation on images. The previous concerns regarding this work included lack of ground truth and not enough clarity regarding the method and the metrics presented. The authors have made significant refinements to the manuscript.

The authors have now included a simulation dataset including use cases corresponding to different cell density, RNA density and signal to noise ratio. The authors have shown that their method outperforms the comparative methods using intersection over union scores. The description of the metrics and simulations has also been added to the manuscript. Additionally, the authors have also included NSCLC dataset and the membrane/nuclei segmentation provided with it as ground truth. The authors have again found that their method outperforms the other comparative methods.

The concern regarding the gene pair correlation metric was addressed well by the authors and authors noted a label mistake in one of the axes of the figure. The authors have clarified that their goal was to highlight the reproducibility in gene pair correlation on different biological replicates. High reproducibility corresponds to high similarity between the gene pair correlation across different replicates. Additionally, the authors have also included further downstream analysis with two biological datasets that reproduce the original biological findings. Finally, the authors have now extended their methods input to multiple channels which further increases its performance and usability.

Overall, the authors have addressed the reviewer comments sufficiently and this manuscript should be accepted.

**Reviewer 3**

The revised manuscript has significantly improved. Notably, the new simulation data analysis is well-designed and provides clearer assessment of model performance. Also, using images containing with both nuclei and cell membrane staining is particularly useful for evaluating the accuracy of cell boundary segmentation. These and other additional analyses have addressed most of the concerns I raised originally. However, there are still a number of remaining issues which I hope the authors could further address.

1. The authors compared their method with CellPose, which has two different modes: one using DAPI only and the other including cell membrane information. They treated the segmentation outcome from using both nucleus and cell membrane as ground truth, but they need to show the cell membrane staining image to justify. In fact, the CellPose DAPI mode does not seem to be reliable in segmenting nucleus boundaries (Figures 3-5), similar errors can occur for the other mode as well. Is there any independent manual segmentation outcome that may serve as a better alternative? In the minimum, words of caution need to be provided for caution against over-interpretation.

2. Why does Figure 5 not have a ground-truth panel as in Figures 3 and 4?

3. It is still unclear to me what ‘reproducibility’ means and how it differs from ‘correlation’. The brief description on page 16 seems to suggest these two terms are more or less the same. If so, changing the terminology does not address my original concern regarding the suspiciously high value of correlation.

**Authors’ response**

Reviewer 3:

The revised manuscript has significantly improved. Notably, the new simulation data analysis is well-designed and provides clearer assessment of model performance. Also, using images containing with both nuclei and cell membrane staining is partic- ularly useful for evaluating the accuracy of cell boundary segmentation. These and other additional analyses have addressed most of the concerns I raised originally. However, there are still a number of remaining issues which I hope the authors could further address.

We thank the review for providing positive feedbacks and further comments on improving the quality and clarity of the manuscript. We have addressed these com- ments accordingly in the revised manuscript.

1. The authors compared their method with CellPose, which has two different modes: one using DAPI only and the other including cell membrane information. They treated the segmentation outcome from using both nucleus and cell mem- brane as ground truth, but they need to show the cell membrane staining image to justify. In fact, the CellPose DAPI mode does not seem to be reliable in segmenting nucleus boundaries (Figures 3-5), similar errors can occur for the other mode as well. Is there any independent manual segmentation outcome that may serve as a better alternative? In the minimum, words of caution need to be provided for caution against over-interpretation.

We thank the reviewer for the comment. We have now added example cell mem- brane staining images, as well as Cellpose segmentation based on both nuclear and membrane images as Supplementary Figure S2 in the revised manuscript. These images suggest that Cellpose segmentation based on both nuclear and membrane images, treated as ground truth for the NSCLC dataset, matches well with visually discerned cell boundaries in the membrane images in most cases. Thus, we argue that ground truth for the NSCLC dataset is generally reliable. That being said, we agree with the reviewer that ground truth obtained by Cellpose may not exactly reflect the actual cell boundaries in all cases. Thus, we have added words of caution in the discussion part of the main manuscript, as the reviewer suggested: In addition, the ground truth for NSCLC dataset in this study is computationally derived from Cellpose. Although this ground truth is generally reliable (Supple- mentary Fig. S2), it may not exactly reflect the real cell boundaries and should be interpreted with care.

We did not use independent manual segmentation in this study for two reasons. First, there is no available manual segmentation provided by the group who orig- inally generated the dataset, or by any independent third party. Second, manual segmentation may also contain noisy segmentation labels that will affect the quality of the ground truth. Since there is no clear evidence that the ground truth used in this study (Cellpose segmentation) will bias towards GeneSegNet or any of the competing methods, we still decided to use Cellpose segmentation as ground truth for the NSCLC dataset.

2. Why does Figure 5 not have a ground-truth panel as in Figures 3 and 4?

We thank the reviewer for the comment. Figure 5 corresponds to the hippocampus dataset, which is a real dataset that does not have membrane staining images, and thus do not have ground truth. Figure 3 corresponds to the simulation dataset and the ground truth was simulated. Figure 4 corresponds to the NSCLC dataset and segmentation derived by both nucleus and membrane was used as the ground truth. Thus, Figure 3 and 4 both have ground-truth panels, but Figure 5 does not.

3. It is still unclear to me what ‘reproducibility’ means and how it differs from ‘correlation’. The brief description on page 16 seems to suggest these two terms are more or less the same. If so, changing the terminology does not address my original concern regarding the suspiciously high value of correlation.

We apologize that the descriptions in the previous version of manuscript was still not clear enough. To more clearly demonstrate the process how reproducibil- ity was calculated, we have now added a new Supplementary Figure S3 in the revised manuscript. In this supplmentary figure, we used a cartoon to demonstrate the whole process how the reproducibility was calculated from the gene expression matrix. First, gene-gene correlations were calculated from the gene expression ma- trix using Pearson correlation. Note that the gene-gene correlations calculated can have both positive and negative values, shown as red and blue colors in the gene- gene correlation matrices. Second, the gene-gene correlation matrices were flattened into vectors. Third, reproducibility was calculated as the Pearson correlation be- tween the flattened vectors. Thus, gene-gene correlation was calculated based on the gene expression matrix, and reproducibility was calculated based on the gene-gene correlation, in a hierarchical manner. We believe that the addition of the new sup- plementary figure has more clearly demonstrated the difference between gene-gene correlations and reproducibility.

**3^rd^ round**

**Reviewer 3**

This revised manuscript has addressed all my remaining concerns satisfactorily.
